# New Insights on Metabolic Features of *Bacillus subtilis* Based on Multistrain Genome-Scale Metabolic Modeling

**DOI:** 10.3390/ijms24087091

**Published:** 2023-04-11

**Authors:** Blas Blázquez, David San León, Antonia Rojas, Marta Tortajada, Juan Nogales

**Affiliations:** 1 Department of Systems Biology, Centro Nacional de Biotecnología, CSIC, 28049 Madrid, Spain; bblazquez@cnb.csic.es (B.B.); dsanleon@cnb.csic.es (D.S.L.); 2 Interdisciplinary Platform for Sustainable Plastics towards a Circular Economy-Spanish National Research Council (SusPlast-CSIC), 28040 Madrid, Spain; 3 Archer Daniels Midland, Nutrition, Biopolis S.L. Parc Científic Universitat de València, Carrer del Catedrático Agustín Escardino Benlloch, 9, 46980 Paterna, Spain; antonia.rojas@amd.com (A.R.); marta.tortajada@adm.com (M.T.)

**Keywords:** *Bacillus subtilis*, genome-scale metabolic model, flux balance analysis, multistrain modeling, panphenome

## Abstract

*Bacillus subtilis* is an effective workhorse for the production of many industrial products. The high interest aroused by *B. subtilis* has guided a large metabolic modeling effort of this species. Genome-scale metabolic models (GEMs) are powerful tools for predicting the metabolic capabilities of a given organism. However, high-quality GEMs are required in order to provide accurate predictions. In this work, we construct a high-quality, mostly manually curated genome-scale model for *B. subtilis* (*i*BB1018). The model was validated by means of growth performance and carbon flux distribution and provided significantly more accurate predictions than previous models. *i*BB1018 was able to predict carbon source utilization with great accuracy while identifying up to 28 metabolites as potential novel carbon sources. The constructed model was further used as a tool for the construction of the panphenome of *B. subtilis* as a species, by means of multistrain genome-scale reconstruction. The panphenome space was defined in the context of 183 GEMs representative of 183 *B. subtilis* strains and the array of carbon sources sustaining growth. Our analysis highlights the large metabolic versatility of the species and the important role of the accessory metabolism as a driver of the panphenome, at a species level.

## 1. Introduction

Aside from being the model organism of Firmicutes clade, *Bacillus subtilis* is a biotechnological workhorse for the production of many industrial products, such as enzymes, vitamins, and antibiotics [1]. In recent years, a considerable effort has been aimed at engineering better-performing *B. subtilis* strains, producing high added value industrial products by means of metabolic engineering [2,3]. However, addressing such an ambitious goal often requires advanced approaches beyond classic metabolic engineering [4]. In this context, the complete understanding of microbial metabolic space, its optimization, and rational expansion require holistic approaches accounting for system-level properties. Since mathematical modeling can be used as accurate scaffolds towards this purpose, it is not surprising that modeling approaches have been developed and thoroughly used for optimizing microbial cell factories and to address important biological questions in the last decade [5,6]. Genome-scale metabolic models (GEMs) are mathematical representations of cellular metabolism accounting for the reactions’ stoichiometries and reversibility under the assumption of a metabolic steady state [7]. Their intrinsic simplicity makes these models powerful tools to analyze biological networks at the genome scale. Overall, GEMs have proven to be powerful computational tools for the computation of the metabolic capabilities and phenotype of a given organism, even under perturbations, and can also be used for strain optimization.

To date, *B. subtilis* has been subject to significant metabolic modeling efforts, and various genome-scale metabolic network models of *B. subtilis* are available: *i*YO844 [8], *i*Bsu1103 [9], *i*Bsu1103v2 [2], *i*Bsu1147 [10], and *i*Bsu1144 [11]. The first GEM, *i*YO844, was constructed including phenotyping and gene essentiality data. More recently, this pioneer model has been updated by integrating 17 enzyme kinetics for the central carbon and related pathway reactions, significantly improving model predictions regarding growth rate and flux distribution [12]. Another series of GEMs of *B. subtilis* was constructed using the semi-automatic workflow developed by the SEED Project [9,13,14,15]. The initial model, *i*Bsu1103 (which also includes Gibbs free energy change values for 1403 of the reactions), has been updated several times in the last decade [2,10,11]. The second update, *i*Bsu1103v2, analyzed and fixed the incorrect gene essentiality predictions of its initial version. Finally, the latest model updates (*i*Bsu1147 and *i*Bsu1144) were aiming to optimize the production of relevant added-value compounds including riboflavin, Egl-237, isobutanol, butanodiol, and serine protease. Despite the efforts in the reconstruction of *B. subtilis* metabolism, it has been recently shown that the current models are far from accurately predicting well-known metabolic traits of this strain [16]. Overall, any discrepancies with experimental data were mainly due to incorrect or incomplete annotations, missing reactions and/or pathways, and/or inaccurate formulation of the biomass reaction. Therefore, there is still much room for improvement in the metabolic modeling of this interesting microorganism.

On the other hand, the increasing number of genomes being sequenced is allowing researchers to address the variability across strains within the same species. As more genome sequences become available, substantial differences in genomic content and functions across strains can be identified [17]. Therefore, it is now possible to explore strain-specific variations using approaches such as pangenome analyses [18]. Pangenome studies have highlighted the existence of important differences among strains, thus explaining divergent phenotypes within a single species. However, despite the utility of pangenome-based functional analyses, they do not provide a mechanistic insight into phenotypic potential based on genetic and genomic variability within a species [19,20]. In this context, the possibility of addressing the metabolic modeling of the whole set of strains of one given species becomes, in an optimal computational framework, an opportunity to analyze, from a mechanistic point of view, the phenotypic differences in a bacterial species [21,22,23,24,25,26,27,28]. However, such multistrain modeling requires high-quality GEMs for each reference strain, which are not always available, thus limiting this approach to a low number of species [25,28,29,30].

In this work, we develop a new and high-quality genome-scale model for *B. subtilis* (*i*BB1018) based on the annotations of the SEED project [15]. *i*BB1018 was built based on *i*Bsu1103v2 but subject to a thoroughly manual curation that included the available biochemical and physiological knowledge. The quality of *i*BB1018 was further validated by means of growth performance and carbon flux distribution providing significantly more accurate predictions than previous models. Overall, *i*BB1018 predicts the carbon sources tested with high precision (84%), while it identifies up to 28 metabolites as potential novel carbon sources, thus significantly expanding the known metabolic versatility of *B. subtilis*. Furthermore, this updated *B. subtilis* GEM provides an essential tool to study pan-metabolic capabilities, thus providing clues about the metabolic range of *Bacillus subtilis* as a species. Subsequently, we provide here a multistrain metabolic model of *B. subtilis*.

## 2. Results

### 2.1. Genome-Scale Metabolic Reconstruction

**Figure 1:**
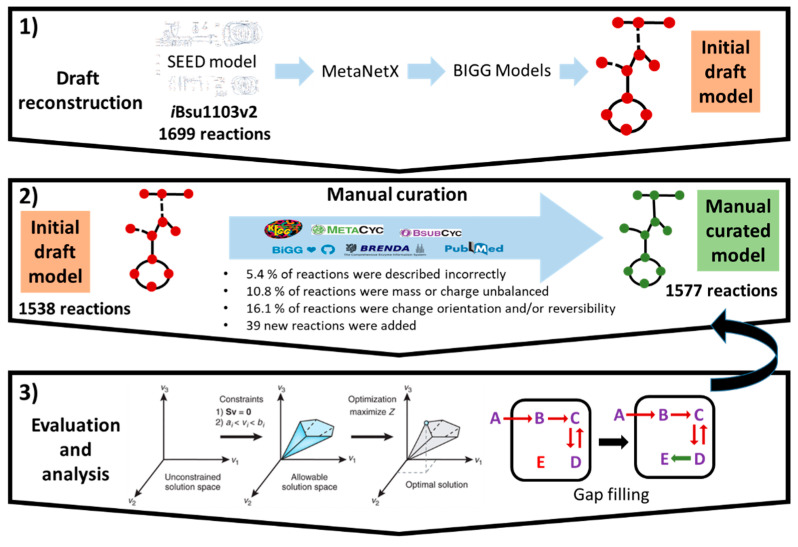
Reconstruction process of *i*BB1018. (1) The initial model was reconstructed using iBsu1103v2 as a template within the framework of MetaNetX and BiGG model databases. (2) An exhaustive manual curation was carried out using bibliographic, biochemical, and metabolic databases. (3) Biomass production was selected as an objective function for gap-filling analysis.

The genome-scale metabolic model *i*BB1018 (Supplementary Additional Files S3 and S4) contains 1577, 1291, and 1018 unique reactions, metabolites, and genes, respectively. The model was created by updating the metabolic content derived from *i*Bsu1103v2 following a procedure that includes three iterative steps (Figure 1). Firstly, an initial draft model was constructed from the SEED-based *i*Bsu1103v2 model. SEED nomenclature was then converted to BiGG annotations using the platform MetaNetX as an intermediate while genes ID were updated according to NCBI (BSU) nomenclature.

Secondly, we carried out an exhaustive revision and manual curation of all the reactions based on the analysis of each Gene–Protein Reaction (GPR) association included in the draft. Therefore, each individual GPR was evaluated based on physiological evidence and information allocated on several databases, such as KEGG [31], MetaCyc [32], and BRENDA [33]. During the manual curation, multiple inconsistencies were found in the draft, which mainly fell into four categories: (1) 5.4% of the reactions initially included in the draft were incorrectly described, in terms of stoichiometry and/or cofactor dependency; (2) 10.8% of the reactions of the initial model were mass or charge unbalanced and were adjusted accordingly; (3) 16.1% of the reactions were modified to change orientation and/or reversibility according to KEGG and MetaCyc databases. Many of these incorrect reactions yielded an unrealistic production of ATP which was not associated with metabolic costs, e.g., pyruvate carboxylase was modified to be irreversible; and (4) 39 new reactions, mostly transporter-related, were added to the model based on physiological and/or genomic data.

Finally, in a third step, we applied a manual gap-filling analysis in order to complete several biosynthetic pathways required to synthesize elements from the biomass reaction. This process was profusely guided by the information available in biological databases and the scientific literature. For instance, it was necessary to add the transporter reactions for D-arabinose, galactitol, and sucrose due to a lack of genes associated with these reactions in the initial draft model.

**Figure 2:**
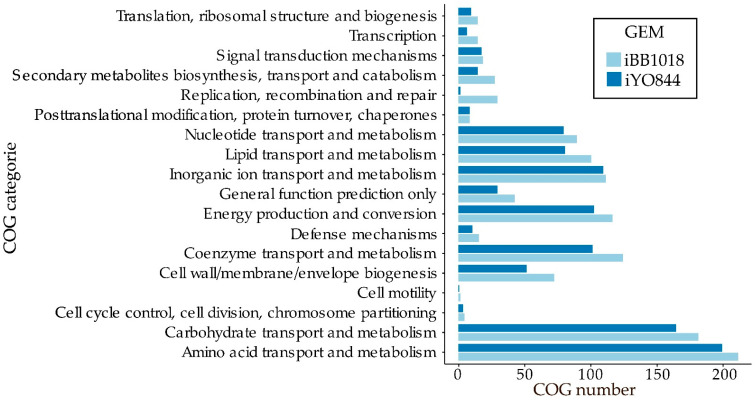
COG comparison between *i*BB1018 and *i*YO844 models. The bars indicate the number of genes associated with a specific COG category.

The metabolic expansion accounted for by *i*BB1018 in comparison with the highly curated model *i*YO844 was highlighted by means of a Clusters of Orthologous Genes (COG) analysis (Figure 2, Table S2). We observed an increase in the number of reactions in almost all the different COGs categories. A noteworthy feature was the significant increase of reactions on carbohydrates transport and metabolism, which led to an increase in the number of new carbon sources used by the model, thus driving a better understanding of the actual metabolic capabilities of *B. subtilis*.

In order to ensure the quality of the *i*BB1018 model, we used the MEMOTE tool [34], a well-known standardized quality control tool for GEMs. The quality evaluation included the missing annotations test, format structuration, searching imbalanced reactions, and searching of errors in stoichiometries. These tests allow the evaluation of the consistency of the metabolic network. The model reached an overall score of 81.09%, which indicates a good model completeness (Figure S1). The consistency of the model scored was 99.8%, which represents the accuracy in stoichiometry, mass and charge balance, metabolite connectivity, and reaction cycles. This analysis confirms that *i*BB1018 is a complete and detailed model. A comparison between the models *i*BB1018, *i*Bsu1103v2, and *i*YO844 by MEMOTE showed high scores for *i*BB1018 and *i*YO844, with 81% and 80%, respectively. On the other hand, the *i*Bsu1103v2 model had the lowest score, 36% (Figure S1, Supplementary Additional File S2).

### 2.2. Growth Rate Performance of iBB1018 Is in Agreement with Several In Vivo Nutritional Scenarios

Large completeness and consistency of a model does not always mean a high-quality model and GEMs need to be validated by assessing their ability to compute physiological states [35]. To validate *i*BB1018, we evaluated its capability predicting well-known metabolic traits of *B. subtilis*, with such growth performance in glucose as the sole carbon source [36]. Aerobic growth was simulated allowing only the uptake of glucose while the upper bound of the rest of the exchange reactions remained unconstrained (Table 1). The performance of *i*BB1018 was further compared with that from previous models including *i*YO844 and the reference SEED model *i*Bsu1103v2. This analysis shows the higher accuracy of *i*BB1018 when compared with previous models. In fact, *i*BB1018 not only predicts the closest growth rate to that reported in vivo, but also a more accurate acetate production. It is noteworthy that while *i*YO844 slightly overestimated acetate secretion, resulting in a significantly lower growth rate, *i*Bsu1103v2 was unable to predict acetate secretion at all, thus overestimating the growth rate.

Since *B. subtilis* is able to grow on additional carbon sources besides glucose, we decided to test the performance of *i*BB1018 on alternative nutritional scenarios in order to complete the model validation. Specifically, we monitored the growth rate of *i*BB1018 using single carbon sources such as citrate, gluconate, and glucose [37], while fixing the riboflavin secretion to experimental data [38,39]. Overall, we found large agreement between in vivo and in silico values, despite model-based predictions slightly overestimating in vivo growth rates in all the carbon sources simulated (Table 2). These discrepancies are within an acceptable error and are often found even in high-quality GEMs [29,30]. They might be explained by: (i) a still incomplete formulation of biomass function, (ii) higher condition-specific energy maintenance requirements not accounted for in the current reconstruction, and (iii) missing adaptation to these alternative compounds as primary carbon sources [35]. Furthermore, these discrepancies may also refer to an incomplete in vivo phenotyping of the strain, for example, not having taken into account possible metabolic byproducts such lactate and pyruvate or the secretion of partially oxidized metabolites.

**Figure 3:**
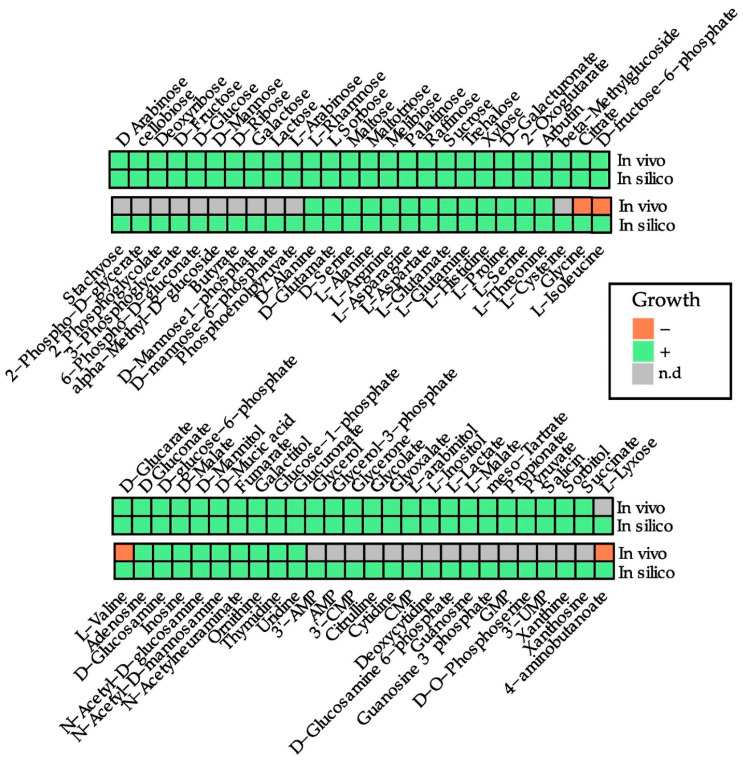
In vivo growth rates based on Biolog data from the literature [8,40,41,42] and in silico carbon sources predicted for *B. subtilis* using the *i*BB1018 model under aerobic conditions. Only carbon sources with positive growth in vivo or in silico are included. The uptake of each potential carbon source was limited to 10 mmol/gDW.h. n.d.: not determinate.

Once *i*BB1018 was validated applying experimental data from the literature, the capability of the in silico model *i*BB1018 to predict aerobic growth on various carbon sources was determined (Figure 3, Table S3 in Supplementary Additional File S1). The in silico computations were compared against experimental data from the literature [8,40,41,42]. Growth on different substrates was simulated by fixing their specific uptake rates to 10 mmol g^−1^ h^−1^ under aerobic conditions in minimal media. *i*BB1018 was able to correctly predict the growth of the bacterium in 80 carbon sources. The model also suggests 28 new compounds as potential carbon sources, including D-xylose or stachyose. This was possible thanks to the expansion of the network, with manual gap filling of reactions involved in the transport of metabolites. Network gap analysis found metabolites that were either consumed only or produced only, and therefore broke the material balance on the metabolites. The candidate metabolites were identified, and reactions were analyzed and filled. Although in silico predictions generally agreed with experimental data, this model also erroneously indicated growth for four carbon sources (4-aminobutanoate, glycine, L-isoleucine, and L-valine), as previous *i*Bsu1103v2 and *i*YO844 models did (Figure 3). Such extended inaccurate predictions argue in favor of a lack of adaptation of *B. subtilis* to these alternative compounds as primary carbon sources, as has been discussed previously [28].

### 2.3. iBB1018 Exhibits Superior Performance Predicting Carbon Flux Distribution Than Previous Models

To further quantitatively evaluate the predictive capabilities of *i*BB1018, we performed a careful analysis of carbon flux distribution using glucose as a single carbon and energy source under aerobic conditions. Subsequently, we compared the fluxes predicted with those experimentally reported [43]. Fluxes prediction using *i*YO844 and *i*Bsu1103v2 were included in the comparison in order to benchmark the performance of these previous models. Overall, this analysis highlights that the manually curated models (*i*YO844 and *i*BB1018) can predict experimentally reported metabolic fluxes with more accuracy (Figure S2, Table S1). In contrast, the automatically constructed *i*Bsu1103v2 yields significantly less accurate fluxes’ predictions.

**Figure 4:**
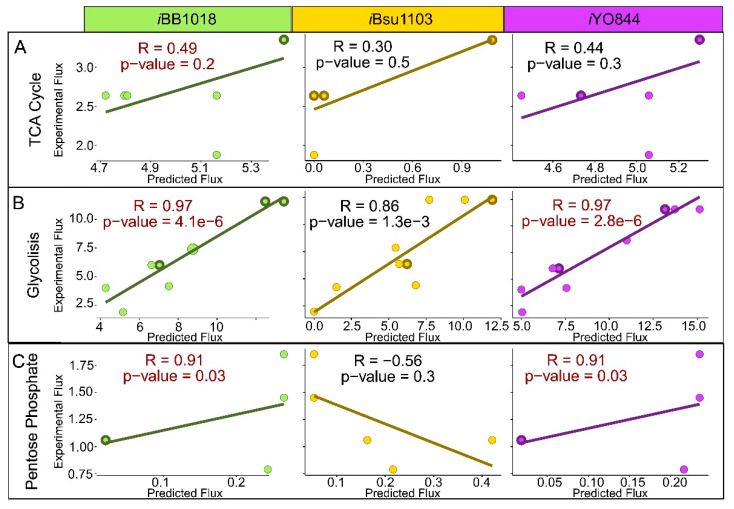
Correlation plot between experimental fluxes from the literature [43] and in silico fluxes predicted by *i*BB1018, *i*Bsu1103v2, and *i*YO844 models for TCA cycle (**A**), glycolysis (**B**), and pentose phosphate pathway (**C**). All the represented fluxes are measured in mmol gDW^−1^ h^−1^. The dots with wider borders contain overlapping values.

When focused on glycolysis, the three models exhibit good agreement with experimental data, despite *i*Bsu1103v2’s slightly underestimated flux through the late pathway. In the case of pentose phosphate pathways, the flux predictions are underestimated, even though the performance of the manually curated models is slightly better in terms of flow correlation. The detailed analysis of the TCA cycle shows that *i*BB1018 and *i*YO844 predicted net flux through all the reactions of TCA. In contrast, *i*Bsu1103v2 significantly underutilizes TCA, predicting null flux through five of the eight reactions involved in the cycle. In addition, the only three active reactions presented underestimated fluxes. A correlation plot was used to compare the models (Figure 4), showing low values for all the models and presenting the model *i*BBb1018 with the highest correlation (R = 0.49, *p*-value 0.2). The *i*Bsu1103v2 model shows, as expected, the lowest correlation values (Figure 4A).

### 2.4. Multistrain Modeling of B. subtilis as Species

Multistrain genome-scale metabolic models can be used to study metabolic diversity and speciation [44]. In order to analyze the phenotypic potential of *B. subtilis,* as species, we first computed the pangenome of *B. subtilis* and, by using the high-quality model *i*BB1018 as a template, we further addressed the multistrain metabolic modeling of the whole species [28].

The pangenome of 184 *B. subtilis* strains was addressed by performing a homology analysis in order to identify orthologous genes among the analyzed strains. We noted a large variability, in terms of number of genes, among the different genomes, ranging from 2623 (CP029052, *B. subtilis* BS155) to 7044 (AP012495, *B. subtilis* BEST7613) (Figure S3, Table S4). Interestingly, the second-largest genome including 4191 genes from *B. subtilis* BEST7613 was identified as a chimera genome harboring an important fragment of the genome of the cyanobacterium *Synechocystis* strain PCC6803 [45]; thus, it was discarded in order to avoid potential errors in the downstream analysis.

**Figure 5:**
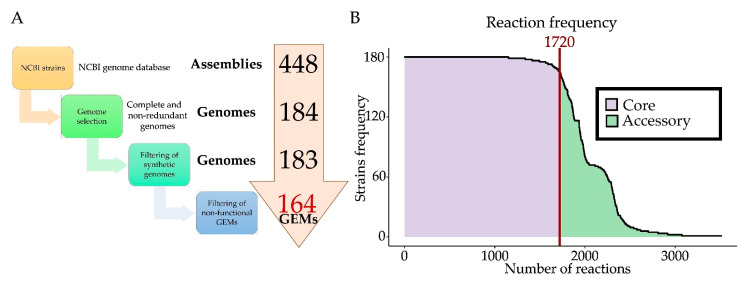
(**A**) Schematic representation of the multiple filtering performed to calculate the final GEMs. (**B**) Reaction frequency among the selected *B. subtilis* strains. The purple area represents core metabolism and green represents accessory metabolism.

Pangenomes make reference to the entire set of genes from all the strains within a clade. Therefore, we defined the core pangenome as that including those genes present in a high percentage of strains (>95%), while the accessory pangenome was defined as that representing unique or shared functions by a reduced group of strains (Figure 5 and Figure S3). While the pangenome is useful in terms of comparative analysis, for modeling purposes, the panphenome is more useful since it is the set of all the in silico metabolic phenotypes displayed by the collection of *B. subtilis* GEMs constructed. Subsequently, we used the estimated pangenome to compute the panphenome of *B. subtilis.* For this, we filtered metabolic genes using the *i*BB1018 and BiGG database as a source of metabolic functions (Methods) and, subsequently, the list of metabolic genes for each strain was reduced to those with any known metabolic function. Interestingly, we found a more homogeneous number of genes in each strain (between 900 and 1200 genes), which strongly suggests that metabolism is highly conserved in contrast to other functionalities. The computed panphenome was finally used as basic metabolic information to construct strain-specific GEMs for each strain analyzed. The GEMs were automatically gap-filled followed by semi-manual curation to include metabolic information other than that included in the core panphenome. A total of 183 strain-specific GEMs were constructed, covering 2324 of the 13,398 gene families present in the calculated pangenome (17.3%). Most of the sequences not included into the GEMs were not annotated or failed to show any known function (35%).

**Figure 6:**
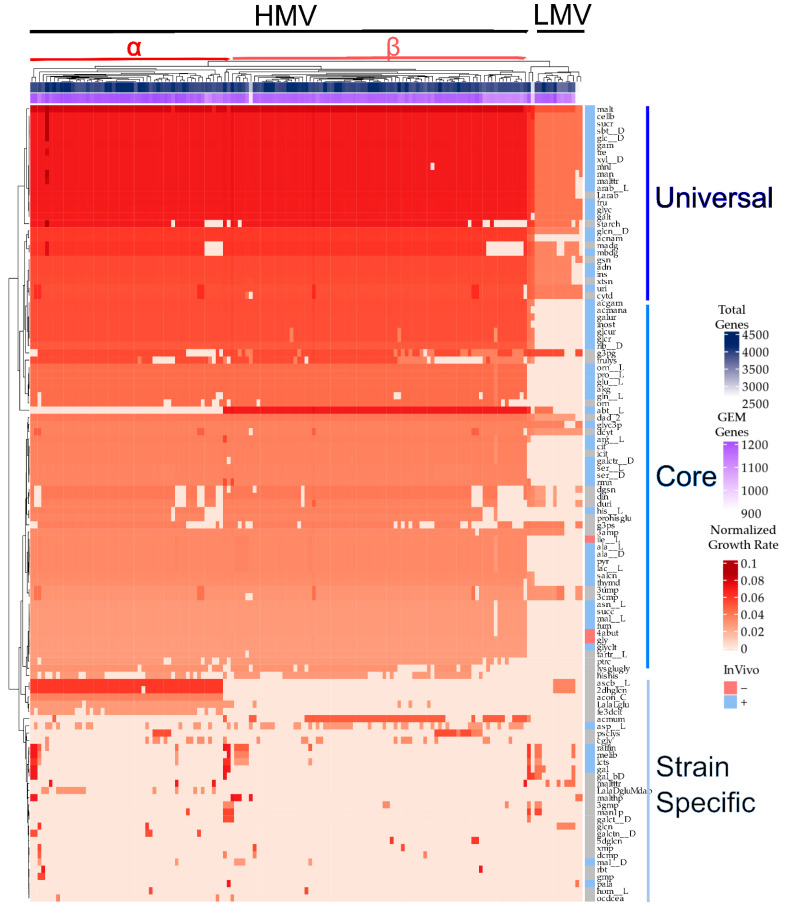
Heatmap with the panphenome growth rates across multiple carbon sources. The top color bars indicate the total number of genes per genome (blue) and the total genes included in the GEMs (purple). The right bar indicates if the carbon source was tested in Biolog with *B. subtilis* 168 and the growth result in vivo. The data were hierarchically clustered with the “manhattan” distance method. The α and β symbols represent HMV subgroups. The universal class contains carbon sources with more than 90% of the strains with growth, the core class contains between 30% and 90%, and the strain-specific class contains less than 30%. The *B. subtilis* strain names are not shown, and the carbon sources are identified with the reduced BiGG namespace IDs to facilitate the visualization. Growth rates were normalized with the number of carbons of each carbon source metabolite.

To validate this multistrain modeling and gain further insights in the phenotypic features of *B. subtilis*, strain-specific GEMs were used to compute those carbon sources supporting growth in the analyzed strains. (Figure 6, Table S5). This analysis unveiled a small set of 18 incomplete and non-functional models which still need additional gap filling or manual curation steps; therefore, only 165 GEMs were further analyzed. Overall, the functional analysis of *B. subtilis* strains based on their cognate GEMs allows the classification of the analyzed strains in two main groups: (i) High Metabolically Versatile (HMV) strains and (ii) Low Metabolically Versatile (LMV) strains (Figure 6). HMV strains show the ability for growth on a large variety of carbon sources, including mono and polysaccharides, amino acids, nucleotides, organic acids, and TCA intermediate, among others. In contrast, LMV strains exhibit a reduced catabolic space, being limited to mono and polysaccharides and a few nucleotides. It is noteworthy that two of the strains identified as LMV, i.e., *B. subtilis* strain PS38 (CP016789) and PG10 (CP016788), were subject to synthetic genome reduction in the context of minimal genome identification [46], therefore explaining, to a great extent, the low metabolic versatility computed. HMV strains could be additionally classified into two subgroups, including HMVα which exhibits growth efficiently in ascorbic acid, 2-dehydro-D-gluconate, Cis-aconitate, and L-alanine-L-glutamate but not on L-arabinitol, and HMVβ which exhibits the opposite phenotype.

Regarding carbon sources, we identified a group of selected monosaccharides, oligosaccharides, and polysaccharides, such as xylose, fructose, and starch, as universal carbon sources (>90% strains with growth) that were able to sustain the growth of most of the *B. subtilis* strains. Interestingly, such carbon sources provided the highest growth rate computed, strongly suggesting optimal carbon sources for *B. subtilis* irrespective of the particular strain being analyzed. A second group of carbon sources that included additional mono and polysaccharides, amino acids, nucleotides, and organic acids were used for a large number of *B. subtilis* strains, constituting a second core carbon source for this species. Finally, we identified an important variety of carbon sources providing growth to only a low number of strains (less than 50); therefore, we consider these compounds as strain-specific carbon sources.

## 3. Discussion

*Bacillus subtili*s is an important microorganism used for biotechnological processes which have triggered a large effort of metabolic modeling in order to construct accurate and predictive computational platforms towards biotechnology endeavors. However, available in silico GEMs of *B. subtilis* are far away, in term of completeness and predictive potential, from equivalent models of other well-known and widely used microbial cell factories such as *Escherichia coli* [29], *Pseudomonas putida* [30], and *Saccharomyces cerevisiae* [47]. In this study, we addressed this challenge and presented an upgraded genome-scale model for *B. subtilis* (*i*BB1018). *i*BB1018 presents higher metabolic completeness and predictive capabilities than previously published *B. subtilis* GEMs, and scores as the highest-quality GEM when analyzed with the MEMOTE tool [34]. The final model was highly validated against reported data and was used for an in-depth analysis of *B. subtilis* metabolism exhibiting a high grade of accuracy in terms of carbon source utilization, growth rate, and flux distribution predictions under various nutritional scenarios.

Once a high-quality GEM exists, its contents (e.g., genes, metabolites, and reactions) can be straightforwardly mapped onto a closely related strain in a species. Following this, a multistrain approach can be integrated with genome-scale modeling in order to gain novel insights underlying the variability of phenotypes. This approach enables panphenome analysis, thus empowering species-wide comparative systems biology that has been applied to well-known species in a variety of studies, mainly involving the assessment of metabolic potential. *E. coli* was the first species subject to multistrain modeling following its high number of GEMs. An initial study by Monk and colleagues addressed the modeling of 55 fully sequenced *E. coli* strains identifying strain-specific auxotrophies and substrate preferences among the set of strains [21]. Extending this analysis, a new multistrain effort included 1200 *E. coli* strains and demonstrated a large variability both in gene content and function within the species. The computed panphenome was further used to construct a robust classification tree for determination between extra-intestinal and intra-intestinal pathogens [29]. Following the *E. coli* example, multistrain modeling has been applied to compute the panphenome of other bacteria having high-quality GEMs available. For instance, 450 *Salmonella* strains were modeled from various serovars to show that metabolic capabilities can be used to discriminate these serovars [25]. Additionally, and taking advantage of high-quality GEMs, environmental and biotechnologically relevant bacteria such as *P. putida* were subject to multistrain modeling, showing that the large metabolic versatility described for many *P. putida* strains is a common feature of the whole species [30].

Despite the large interest in *B. subtilis*, its multistrain modeling has remained elusive dues to the lack of high-quality GEMs to be used as guide. Here, by using *i*BB1018 as a high-quality template, we have addressed the modeling of 184 *B. subtilis* strains whose genomes were available at the time this study started. Overall, we were able to construct a total of 165 functional GEMs which define a large panphenome space in terms of a carbon source supporting growth (Figure 6). Overall, we found a large phenotypic homogeneity. Most of the strains could be classified as high metabolically versatile according to the number of carbon sources they were capable of using. With some exceptions, we found that 95% of the strains were able to use up to 72% of the carbon source analyzed as a sole carbon and energy source. Interestingly, we identified that a large number of carbon sources (28%) were only used by a few strains (less than 50), highlighting the role of the accessory metabolism as the driving force of the panphenome at a species level [48]. In addition to the intrinsic value of the panphenome identified in this study, the collection of *B. subtilis* GEMs provided here constitutes an intangible value which can be leveraged as high-quality guidance in future metabolic studies of this valuable bacteria species.

## 4. Materials and Methods

### 4.1. Model Reconstruction and Analysis

*i*BB1018 was reconstructed by refining and updating the previous genome-scale model for *B. subtilis*. The metabolic network was reconstructed using standardized protocols for metabolic reconstruction [35,49]. An initial draft reconstruction was generated from the annotated genome of *B. subtilis* str. 168 (GenBank number: AL009126.3) and the metabolic content from model *i*Bsu1103v2 based on SEED annotations. The draft model was converted to BiGG annotations through MetaNetX [50]. The final draft was subjected to manual curation of the metabolic information in order to remove potential inconsistencies and to connect the network as much as possible, using metabolic and GEMs databases including KEGG [31], MetaCyc [32], BRENDA [33], and BiGG [51]. Transport reactions were added by using the TransportDB database [52]. To improve the functionality of the model, gap filling was performed using the RAVEN function *fillGaps* to ensure that the model could grow in silico. Moreover, a manual gap-filling step was performed in order to connect the network as much as possible and remove potential inconsistencies or close death-end metabolites. Finally, it was confirmed that all reactions were balanced, and all metabolites were annotated with their charge and location (_c, cytosol; _e, extracellular space).

A robust correlation method, biweight midcorrelation [53] implemented in the WGCNA R package [54], was used to compare the samples. This non-parametric, median-based method was chosen because the datasets do not follow a normal distribution and it is less sensitive to outliers. The plots were generated with the R package ggpubr [55].

To compare the performance between the three models, MEMOTE [34] was executed with the function “diff”.

For the comparison of COGs between models, the genes from the *i*BB1018 and *i*YO844 models were linked to COGs by using the NCBI COG database version 2020 (https://www.ncbi.nlm.nih.gov/research/cog, accessed on 22 December 2022) (Table S2, Supplementary Additional File S1). The genes were aggregated into COG categories to identify the improvements in high-level systems and in the case associated with multiple COG categories, they were counted multiple times.

### 4.2. Flux Balance Analysis (FBA)

Flux Balance Analysis (FBA) was used to evaluate the biomass production and to predict flux distributions once the biomass reaction was fixed as the objective function (BOF, Biomass Objective Function). The foundations and applications of FBA have been reviewed elsewhere [56]. The growth prediction when executing FBA was the growth rate (h^−1^) predicted under the minimal M9 media, as previously described [30]. All the exchange reactions were sequentially tested in minimal media as potential carbon and nitrogen sources, using an uptake of −10 mmol gDW^−1^ h^−1^ to avoid limitations. Regarding carbon sources, growth rate was further normalized considering the number of carbons of each compound in both oxic and anoxic conditions. The COBRA ToolBox v.3.0 [57] running on the MatLab platform was used for FBA simulations.

### 4.3. Multistrain

All assemblies of diverse strains of *B. subtilis* were downloaded from the NCBI database (488 downloaded assemblies). Only the complete assemblies (NCBI completeness class) with gene annotation and proteome availability were used for this analysis. To reduce redundancy, only non-redundant genomes at the proteome level were selected, with 184 strains in total. The final genome dataset contained 184 genomes of *B. subtilis*. To perform multistrain models, the model developed for this work was used as a template and, following the protocol designed by Norsigian [28], the models of other strains were created. To identify the multiple carbon sources used by the strains, in-house python scripts were used.

Multi-gap filling was run over the single-strain models without growth in any carbon source to identify essential genes from the metabolic information, with the results combined with the homology results. For this analysis, the minimum percentage of homology used to mark a gene as an ortholog of *B. subtilis* 168 was 70%, but this threshold was relaxed when the number of genes of strains with a percentage of homology over 40% was present in more than 75% of strains. In this case, the threshold decreased to 40% of homology.

The genes without homology against the template were compared to the total BiGG gene datasets to identify possible homologs in other species and models. The involved reactions were included in the final models.

The carbon source analysis over the *B. subtilis* strains GEMs were driven by the FBA implementation of COBRApy v0.20. The uptakes were normalized based on the number of carbons. The code and the multistrain GEMs can be accessed in the following GitHub repository (https://github.com/SBGlab/Bacillus_Subtilis_multistrain_GEM).

## Figures and Tables

**Table 1:** Parameters of *B. subtilis* 168 growing in glucose in vivo and in silico. Constraints used to feed the model are underlined.

	Glucose Uptake mmol g^−1^ h^−1^	Oxygen Uptake mmol g^−1^ h^−1^	Growth Rate h^−1^	Acetate Production mmol g^−1^ h^−1^
In vivo [36]	8.71 ± 0.64	18	0.67 ± 0.02	4.28 ± 0.29
*i*BB1018	8.71	18	0.69	4.03
*i*Bsu1103v2	8.71	18	1.27	0
*i*YO844	8.71	18	0.61	5.53

**Table 2:** Validation of growth performance of *i*BB1018 under various carbon source and production scenarios. Constraints used to feed the model are underlined.

Carbon Source Substrate ^a^ Uptake (mmol g^−1^ h^−1^)	In Vivo Riboflavin Secretion (mmol g^−1^ h^−1^)	In Vivo Growth Rate (h^−1^)	In Silico Growth Rate (h^−1^)
v*glc* = 1.55; v*cit* = 0.5	0.0173	0.10	0.12
v*glc* = 1.55; v*glcn_D* = 0.6	0.0258	0.12	0.13
v*glc* = 1.7	0.0181	0.10	0.10
v*glc* = 3.2	0.0210	0.20	0.24
v*glc* = 4.7	0.0231	0.30	0.39
v*glc* = 6.2	0.0255	0.40	0.54

^a^ glc: D-glucose; cit: citrate; glcn_D: D-gluconate.

## Data Availability

Publicly available datasets were analyzed in this study. This data can be found here: https://github.com/SBGlab/Bacillus_Subtilis_multistrain_GEM.

## Supplementary Materials

The supplementary materials are available online at https://www.mdpi.com/article/10.3390/ijms24087091/s1.
