# Supplementary material for: New Insights on Metabolic Features of Bacillus subtilis Based on Multistrain Genome-Scale Metabolic Modeling
Source: Int J Mol Sci. 2023 Apr 11;24(8):7091. doi: 10.3390/ijms24087091 (PMC10138676; doi:10.3390/ijms24087091)
Supplement: Supplementary file 1 [file ijms-24-07091-s001.zip › Supplemental material/Additional File S1.docx]

**Appendix S1**


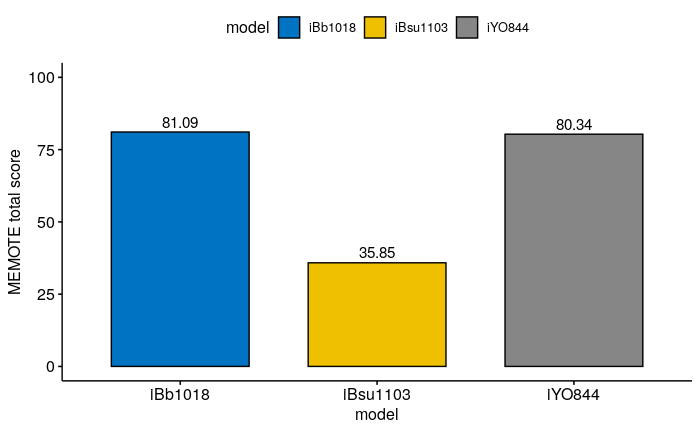


**Figure S1.** MEMOTE total score for the model *i*Bb1018, *i*Bsu1103v2 and *i*YO844.

**Table S2.** Experimental fluxes of the central carbon reactions for *B. subtilis* [1] and predicted fluxes from the models *i*BB1018, *i*Bsu1103v2 and *i*YO844.

| **Reaction**  **names** | **EXP FLUX [1]** | *i***BB1018** | *i***Bsu1103v2** | *i***YO844** |
| --- | --- | --- | --- | --- |
| EX_glc_bD(e) | -7.71 | -7.71 | -7.71 | -7.71 |
| EX_o2(e) | -18.00 | -18.00 | -18.00 | -18.00 |
| BS_Biomass_10 | 0.610 | 0.645 | 1.111 | 0.578 |
| EX_ac(e) | 3.36 | 1.803 | 0.000 | 3.127 |
| CS | 3.353 | 5.429 | 1.085 | 5.299 |
| ACONT | 3.353 | 5.429 | 1.085 | 5.299 |
| ICDHyr | 3.353 | 5.429 | 1.085 | 5.299 |
| AKGDH | 2.638 | 4.795 | 0.065 | 4.734 |
| SUCOAS | 2.638 | 4.721 | 0.061 | 4.450 |
| SUCD1 | 2.638 | 4.807 | 0.000 | 4.734 |
| FUM | 2.638 | 5.162 | 0.000 | 5.057 |
| MDH | 1.878 | 5.162 | 0.000 | 5.057 |
| PGI | 4.159 | 7.519 | 6.802 | 7.565 |
| PFK | 6.005 | 7.039 | 6.218 | 7.140 |
| FBA | 6.005 | 7.039 | 6.218 | 7.140 |
| TPI | 6.005 | 6.629 | 5.697 | 6.790 |
| GAPD_NAD | 11.577 | 13.445 | 11.920 | 15.140 |
| PGK | 11.577 | 13.445 | 11.920 | 13.735 |
| PGM | 11.577 | 12.485 | 7.710 | 13.172 |
| ENO | 11.577 | 12.485 | 10.081 | 13.172 |
| PYK | 4 | 4.265 | 1.499 | 5.008 |
| PDH | 7.405 | 8.757 | 5.454 | 9.555 |
| G6PDH2r | 3.29 | 0.000 | 0.625 | 0.000 |
| RPE | 1.85 | 0.265 | 0.053 | 0.235 |
| RPI | 1.45 | 0.265 | 0.053 | 0.235 |
| TKT1 | 1.06 | 0.021 | 0.163 | 0.019 |
| TKT2 | 0.79 | 0.243 | 0.216 | 0.216 |
| TAL | 1.06 | 0.021 | 0.421 | 0.019 |
| PTAr | 3.37 | 1.488 | 0.000 | 2.880 |
| ACKr | 3.37 | 1.488 | 0.000 | 2.880 |

**Figure S2.** Experimental and predicted fluxes of the central carbon reactions for B. subtilis. Predictions using *i*Bb1018 (red bars), *i*Bsu1103v2 (purple bars) and *i*YO844 (green bars) for growth rate, acetate secretion and fluxes through the reactions of glycolysis, TCA cycle and pentose phosphate pathway.

**Table S3.** List of carbon sources predicted to not be used by *Bacillus subtilis* using the *i*BB1018 model in aerobic conditions. The uptake of each potential carbon source was limited to 10 mmol / gDW.h.

| **Carbon Sources** | | | | | |
| --- | --- | --- | --- | --- | --- |
|  | ***in silico*** |  | ***in silico*** |  | ***in silico*** |
| Saligenin | - | Aminoethanol | - | Niacin | - |
| 4-Hydroxy-benzyl alcohol | - | ethanesulfonate | - | Phenylacetaldehyde | - |
| 5-Methylthio-D-ribose | - | Ethanol | - | L-Phenylalanine | - |
| Acetate | - | Ferrichrome | - | Protoheme | - |
| Adenine exchange | - | Ferroxamine | - | (R)-Pantothenate | - |
| beta-Alanine | - | Formate | - | Prephenate | - |
| ala-L-asp-L | - | gamma-butyrobetaine | - | Puromycin | - |
| ala-L-glu-L | - | gly-asn | - | Pyridoxal | - |
| Ala-His | - | gly-asp | - | Riboflavin | - |
| Ala-Leu | - | gly-gln | - | D-Gulitol | - |
| ala-L-Thr-L | - | gly-glu | - | Shikimate | - |
| Allantoin | - | gly-met | - | Sulfoacetate | - |
| Arsenobetaine | - | gly-pro | - | Taurine | - |
| R R 2 3 Butanediol | - | Glycogen | - | Thioglycolate | - |
| Biotin | - | Heme D | - | Thiamin | - |
| Carbamoylphosphate | - | hexanesulfonate | - | L-Tyrosine | - |
| Cys-Gly | - | Quinol | - | UMP | - |
| Choline | - | L-alanylglycine | - | Uracil | - |
| Chorismate | - | Lanthionine | - | Urate | - |
| Carnitine | - | L-Cysteate | - | Urea | - |
| Cytosine | - | L-Leucine | - | Acetoin | - |
| L-Cystine | - | Lipoate | - | Thymine | - |
| Deoxyadenosine | - | L-Lysine | - | Methanol | - |
| 5'-Deoxyadenosine | - | L-Methionine | - | Acetoacetate | - |
| Dextrin | - | Butyro-betaine | - | 2-Oxobutanoate | - |
| Thymidine 5'-monophosphate | - | MOPS | - | Trimetaphosphate | - |
| Ectoine | - | methanesulfonate | - |  | - |


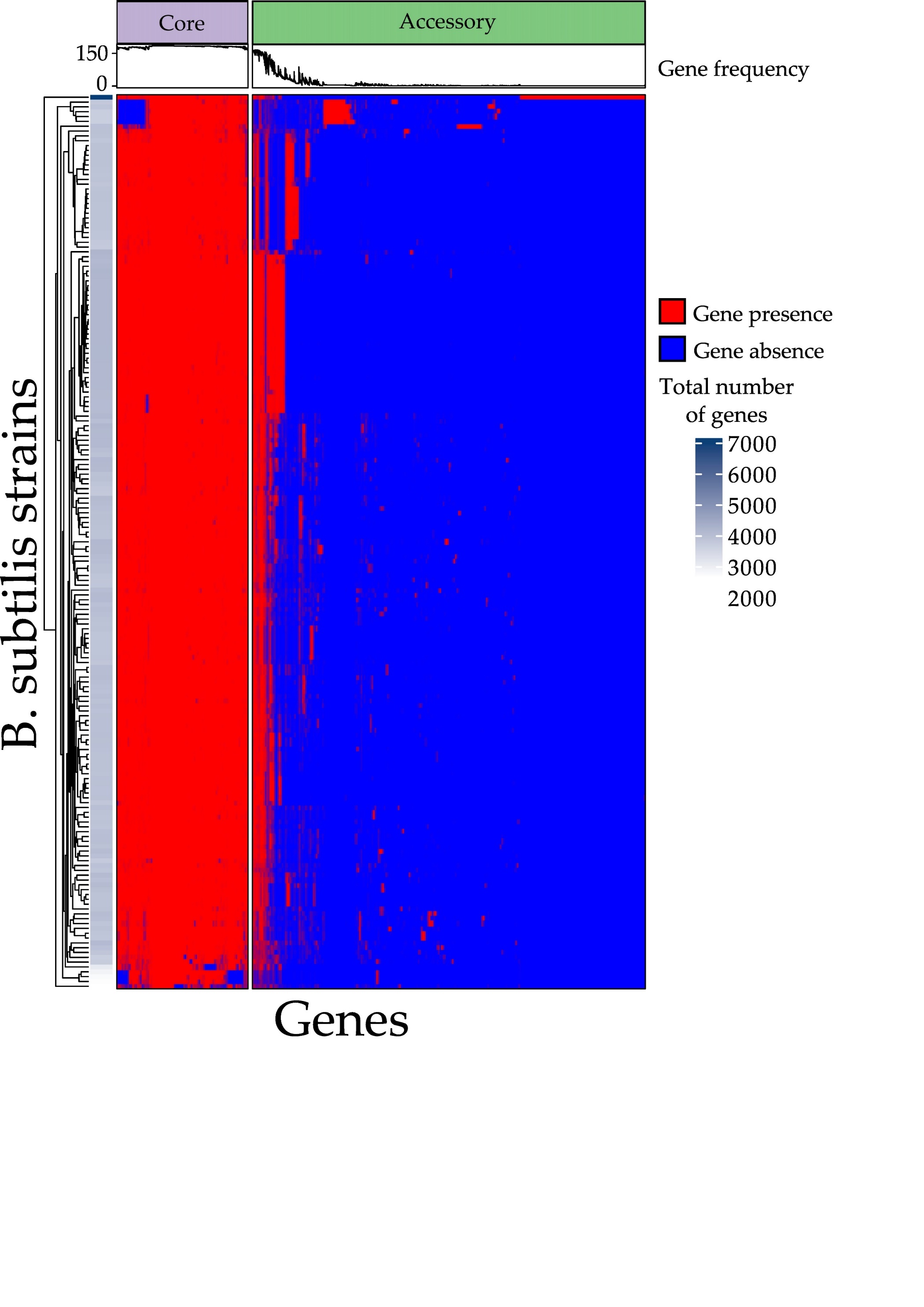
**Figure S3.** Heatmap of *B. subtilis* pangenome. 184 strains are represented on the columns and the genes are represented on the columns. The color represents the presence or absence of gene in the correspondence strains. The upper line diagram represents the frequency of the presence of the genes in the total number of strains.

References

1. Chubukov, V.; Uhr, M.; Le Chat, L.; Kleijn, R.J.; Jules, M.; Link, H.; Aymerich, S.; Stelling, J.; Sauer, U. Transcriptional regulation is insufficient to explain substrate-induced flux changes in *Bacillus subtilis*. *Mol. Syst. Biol.* **2013**, *9*, doi:10.1038/msb.2013.66.
