# Supplementary material for: New Insights on Metabolic Features of Bacillus subtilis Based on Multistrain Genome-Scale Metabolic Modeling
Source: Int J Mol Sci. 2023 Apr 11;24(8):7091. doi: 10.3390/ijms24087091 (PMC10138676; doi:10.3390/ijms24087091)
Supplement: Supplementary file 1 [file ijms-24-07091-s001.zip › Supplemental material/Additional File S2.pdf]

Independent Section

Contains tests that are independent of the class of modeled organism, a model's complexity or types of identifiers that are used to describe its components. Parameterization or initialization of the network is not required. See readme for more details.

Consistency

Compared Models	iBb1018.xml	iBsu1103.xml	iYO844.xml
Stoichiometric Consistency	100.0%	100.0%	100.0%
Mass Balance	92.4%	0.0%	94.4%
Charge Balance	92.6%	99.5%	98.9%
Metabolite Connectivity	100.0%	99.7%	100.0%
Unbounded Flux In Default Medium	0.0%	0.0%	0.0%
Sub Total	84%	71%	85%

Annotation - Metabolites

Compared Models	iBb1018.xml	iBsu1103.xml	iYO844.xml
Presence of Metabolite Annotation	100.0%	0.0%	100.0%
Metabolite Annotations Per Database			Info
pubchem.compound	0.0%	0.0%	0.0%
kegg.compound	67.5%	0.0%	79.7%
seed.compound	72.8%	0.0%	90.1%
inchikey	68.9%	0.0%	0.0%
inchi	0.0%	0.0%	0.0%
chebi	70.9%	0.0%	87.9%
hmdb	53.3%	0.0%	64.9%
reactome	35.6%	0.0%	0.0%
metanetx.chemical	76.0%	0.0%	100.0%
bigg.metabolite	100.0%	0.0%	100.0%
biocyc	69.2%	0.0%	81.5%
Metabolite Annotation Conformity Per Database			Info
pubchem.compound	0.0%	0.0%	0.0%
kegg.compound	100.0%	0.0%	100.0%
seed.compound	100.0%	0.0%	100.0%
inchikey	100.0%	0.0%	0.0%
inchi	0.0%	0.0%	0.0%
chebi	100.0%	0.0%	100.0%
hmdb	100.0%	0.0%	100.0%
reactome	100.0%	0.0%	0.0%
metanetx.chemical	100.0%	0.0%	100.0%
bigg.metabolite	100.0%	0.0%	100.0%
biocyc	100.0%	0.0%	100.0%
Uniform Metabolite Identifier Namespace	100.0%	100.0%	100.0%
Sub Total	84%	25%	80%

Annotation - Reactions

Compared Models	iBb1018.xml	iBsu1103.xml	iYO844.xml
Presence of Reaction Annotation	100.0%	12.7%	100.0%
Reaction Annotations Per Database			Info
rhea	43.5%	0.0%	50.8%
kegg.reaction	36.8%	0.0%	40.7%
seed.reaction	64.2%	0.0%	78.5%
metanetx.reaction	74.4%	0.0%	100.0%
bigg.reaction	100.0%	0.0%	100.0%
reactome	14.2%	0.0%	0.0%
ec-code	44.7%	0.0%	50.7%
brenda	0.0%	0.0%	0.0%
biocyc	42.5%	0.0%	48.9%
Reaction Annotation Conformity Per Database			Info
rhea	98.2%	0.0%	97.9%
kegg.reaction	100.0%	0.0%	100.0%
seed.reaction	100.0%	0.0%	100.0%
metanetx.reaction	100.0%	0.0%	100.0%
bigg.reaction	100.0%	0.0%	100.0%
reactome	100.0%	0.0%	0.0%
ec-code	97.8%	0.0%	98.3%
brenda	0.0%	0.0%	0.0%
biocyc	100.0%	0.0%	100.0%
Uniform Reaction Identifier Namespace	100.0%	100.0%	100.0%
Sub Total	84%	28%	82%

Annotation - Genes

Compared Models	iBb1018.xml	iBsu1103.xml	iYO844.xml
Presence of Gene Annotation	100.0%	0.0%	100.0%
Gene Annotations Per Database			Info
refseq	98.8%	0.0%	0.0%
uniprot	97.9%	0.0%	0.0%
ecogene	0.0%	0.0%	0.0%
kegg.genes	92.7%	0.0%	0.0%
ncbigi	75.0%	0.0%	99.6%
ncbigene	98.8%	0.0%	0.0%
ncbiprotein	98.8%	0.0%	0.0%
ccds	0.0%	0.0%	0.0%
hprd	0.0%	0.0%	0.0%
asap	0.0%	0.0%	0.0%
Gene Annotation Conformity Per Database			Info
refseq	100.0%	0.0%	0.0%
uniprot	100.0%	0.0%	0.0%
ecogene	0.0%	0.0%	0.0%
kegg.genes	7.3%	0.0%	0.0%
ncbigi	25.0%	0.0%	0.4%
ncbigene	100.0%	0.0%	0.0%

Specific Section

Covers general statistics and specific aspects of a metabolic network that are not universally applicable. See readme for more details.

SBML

Compared Models	iBb1018.xml	iBsu1103.xml	iYO844.xml
SBML Level and Version	SBML Level 3 Version 1	SBML Level 2 Version 1	SBML Level 3 Version 1
FBC enabled	true	false	true

Basic Information

Compared Models	iBb1018.xml	iBsu1103.xml	iYO844.xml
Model Identifier	iJN1026	iBsu1121VCorrected	iYO844
Total Metabolites	1,291	1,625	990
Total Reactions	1,580	1,925	1,250
Total Genes	1,020	1,109	844
Total Compartments	2	2	2
Metabolic Coverage	1.55	1.74	1.48
Uncoserved Metabolites	0	0	0
Minimal Inconsistent Net Stoichiometries	0	0	0

Metabolite Information

Compared Models	iBb1018.xml	iBsu1103.xml	iYO844.xml
Unique Metabolites	1,291	1,625	779
Duplicate Metabolites in Identical Compartments	1	0	0
Metabolites without Charge	0	0	0
Metabolites without Formula	0	1,625	0
Medium Components	21	244	13

Reaction Information

Compared Models	iBb1018.xml	iBsu1103.xml	iYO844.xml
Purely Metabolic Reactions	1,103	1,680	769
Purely Metabolic Reactions with Constraints	2	0	3
Transport Reactions	260	0	251
Transport Reactions with Constraints	1	0	1
Reactions With Partially Identical Annotations	0.03	0.00	0.03
Duplicate Reactions	1.00	0.00	0.00
Reactions With Identical Genes	0.56	0.50	0.38

Gene-Protein-Reaction (GPR) Associations

Compared Models	iBb1018.xml	iBsu1103.xml	iYO844.xml
Reactions without GPR	1	244	117
Fraction of Transport Reactions without GPR	0.00	1.00	0.31
Enzyme Complexes	196	199	138

Biomass

Compared Models	iBb1018.xml	iBsu1103.xml	iYO844.xml
Biomass Reactions Identified	3	1	1
Biomass Consistency			Info
bio00006			0.00
BIOMASS_BS_10			1.04
Biomass Production In Default Medium			Info
BiomassRepsol			0.00
BiomassRepsolPrueba			0.69
BiomassRepsolRed			0.69
bio00006			415.29
BIOMASS_BS_10			0.12
Unrealistic Growth Rate In Default Medium			Info
BiomassRepsol			false
BiomassRepsolPrueba			false
BiomassRepsolRed			false
bio00006			true
BIOMASS_BS_10			false
Biomass Production In Complete Medium			Info
BiomassRepsol			0.00
BiomassRepsolPrueba			80.76
BiomassRepsolRed			80.76
bio00006			415.29
BIOMASS_BS_10			157.95
Blocked Biomass Precursors In Default Medium			Info
BiomassRepsol			2
BiomassRepsolPrueba			0
BiomassRepsolRed			0
bio00006			1
BIOMASS_BS_10			0
Blocked Biomass Precursors In Complete Medium			Info
BiomassRepsol			2
BiomassRepsolPrueba			0
BiomassRepsolRed			0
bio00006			1
BIOMASS_BS_10			0
Ratio of Direct Metabolites in Biomass Reaction			Info
BiomassRepsol			0.13
BiomassRepsolPrueba			0.16
BiomassRepsolRed			0.14
bio00006			0.00
BIOMASS_BS_10			0.07
Number of Missing Essential Biomass Precursors			Info
BiomassRepsol			27

hprd	0.0%	0.0%	0.0%
asap	0.0%	0.0%	0.0%
**Sub Total**	66%	0%	37%

Annotation - SBO Terms

Compared Models	iBb1018.xml	iBsu1103.xml	iYO844.xml
Metabolite General SBO Presence	100.0%	0.0%	100.0%
Metabolite SBO:0000247 Presence	100.0%	0.0%	100.0%
Reaction General SBO Presence	100.0%	12.7%	100.0%
Metabolic Reaction SBO:0000176 Presence	99.2%	0.0%	100.0%
Transport Reaction SBO:0000185 Presence	99.2%	Skipped	100.0%
Exchange Reaction SBO:0000627 Presence	100.0%	100.0%	100.0%
Demand Reaction SBO:0000628 Presence	Skipped	Skipped	Skipped
Sink Reactions SBO:0000632 Presence	Skipped	Skipped	Skipped
Gene General SBO Presence	100.0%	0.0%	100.0%
Gene SBO:0000243 Presence	100.0%	0.0%	100.0%
Biomass Reactions SBO:0000629 Presence	100.0%	0.0%	100.0%
**Sub Total**	82%	10%	82%
**Total Score**	81%	36%	80%

Total Score

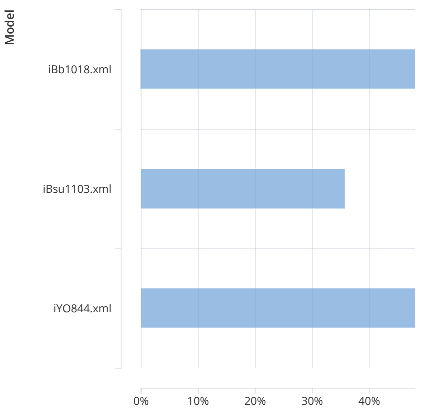

Score per Category

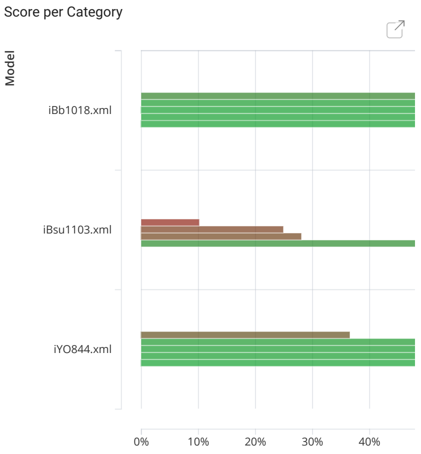

bio00006			30
BIOMASS_BS_10			6

Energy Metabolism

Compared Models	iBb1018.xml	iBsu1103.xml	iYO844.xml
Non-Growth Associated Maintenance Reaction	1	1	1
Growth-associated Maintenance in Biomass Reaction			Info
BiomassRepsol			false
BiomassRepsolPrueba			false
BiomassRepsolRed			false
bio00006			false
BIOMASS_BS_10			true
Number of Reversible Oxygen-Containing Reactions	3	13	3

Network Topology

Compared Models	iBb1018.xml	iBsu1103.xml	iYO844.xml
Universally Blocked Reactions			
Orphan Metabolites	79	58	49
Dead-end Metabolites	93	96	122
Stoichiometrically Balanced Cycles			
Metabolite Production In Complete Medium			
Metabolite Consumption In Complete Medium			

Matrix Conditioning

Compared Models	iBb1018.xml	iBsu1103.xml	iYO844.xml
Ratio Min/Max Non-Zero Coefficients	0.00	0.00	0.00
Independent Conservation Relations	57	71	22
Rank	1234	1554	959
Degrees Of Freedom	346	371	291

Experimental Data Comparison

Compared Models
